# Proteasome Activator Enhances Survival of Huntington's Disease Neuronal Model Cells

**DOI:** 10.1371/journal.pone.0000238

**Published:** 2007-02-28

**Authors:** Hyemyung Seo, Kai-Christian Sonntag, Woori Kim, Elena Cattaneo, Ole Isacson

**Affiliations:** 1 Neuroregeneration Laboratories, Center for Neuroregeneration Research, McLean Hospital and Harvard Medical School, Belmont, Massachusetts, United States of America; 2 Department of Molecular and Life Sciences, Hanyang University, Gyeonggi-do, South Korea; 3 Center on Neurodegenerative Diseases, University of Milan, Milan, Italy; Laboratory of Neurogenetics, National Institutes of Health, United States of America

## Abstract

In patients with Huntington's disease (HD), the proteolytic activity of the ubiquitin proteasome system (UPS) is reduced in the brain and other tissues. The pathological hallmark of HD is the intraneuronal nuclear protein aggregates of mutant huntingtin. We determined how to enhance UPS function and influence catalytic protein degradation and cell survival in HD. Proteasome activators involved in either the ubiquitinated or the non-ubiquitinated proteolysis were overexpressed in HD patients' skin fibroblasts or mutant huntingtin-expressing striatal neurons. Following compromise of the UPS, overexpression of the proteasome activator subunit PA28γ, but not subunit S5a, recovered proteasome function in the HD cells. PA28γ also improved cell viability in mutant huntingtin-expressing striatal neurons exposed to pathological stressors, such as the excitotoxin quinolinic acid and the reversible proteasome inhibitor MG132. These results demonstrate the specific functional enhancements of the UPS that can provide neuroprotection in HD cells.

## Introduction

Huntington's disease (HD) is an adult onset autosomal dominant inherited disease, characterized clinically by a progressive movement and psychiatric disorder. Neuropathologically, HD is associated with neuronal dysfunction and cell death, especially in the caudate-putamen (striatum) region of the brain [1]. HD is caused by mutations increasing the number of CAG repeats in exon 1 of the huntingtin gene (IT15), which is expressed in most cells of the human body [2]. A hallmark of the disease neuropathology is intracellular ubiquitin positive nuclear inclusion bodies of mutated huntingtin [3]–[6]. One potential cause for such abnormal protein aggregation is dysfunction or overloading of the ubiquitin-proteasome system (UPS), which is essential for the clearance of short-lived, mislocated, misfolded, mutated, and damaged proteins in eukaryotic cells [7], [8].

Previously, we discovered that proteasome activities are inhibited in striatum, frontal cortex, cerebellum and substantia nigra of HD patients' brain, and also in non-brain cells such as their skin fibroblasts [9]. In HD patients, we also found increased ubiquitin expression levels, and confirmed decreased mitochondrial complex II–III (MCII–III) enzyme activities in the caudate putamen region of the brain, and decreased brain derived neurotrophic factor (BDNF) protein levels in several brain regions of HD patients [9]. These data indicated that UPS dysfunction may precipitate the critical pathology of the vulnerable medium sized spiny neurons in the striatum [9]. It is therefore of interest therapeutically to study whether improved UPS function can reduce the abnormal protein degradation and increase cell survival in HD.

How does the UPS degrade abnormal proteins? The UPS is a large multisubunit protease assembly, where protein substrates are enzymatically processed at the catalytic sites of the central core chamber of the 20S proteasome [10], [11]. Corresponding to the function of the different subunits of the catalytic core, the activities of the 20S proteasome include (1) chymotrypsin-like (after hydrophobic residues), (2) trypsin-like (after basic residues), or (3) peptidyl-glutamyl preferring hydrolytic (PGPH, after basic residues) activities [12], [13]. When the amounts of intracellular abnormal proteins are increased, this catalytic core can assemble with proteasome activators (PA). There are two types of PA: PA28 subunits for non-ubiquitinated and PA700 subunits for ubiquitinated proteolysis, which can facilitate protein substrate entry and activation of proteasome function [14], [15]. In this report, we first describe the effect of the mutant huntingtin protein on UPS function in different HD relevant cellular systems, and then demonstrate that gene transfer of the PA subunits of the UPS can enhance abnormal protein degradation leading to improved function and survival of cells in HD.

## Results

### Experimental strategy for the modification of the UPS in HD model cells

**Figure 1:**
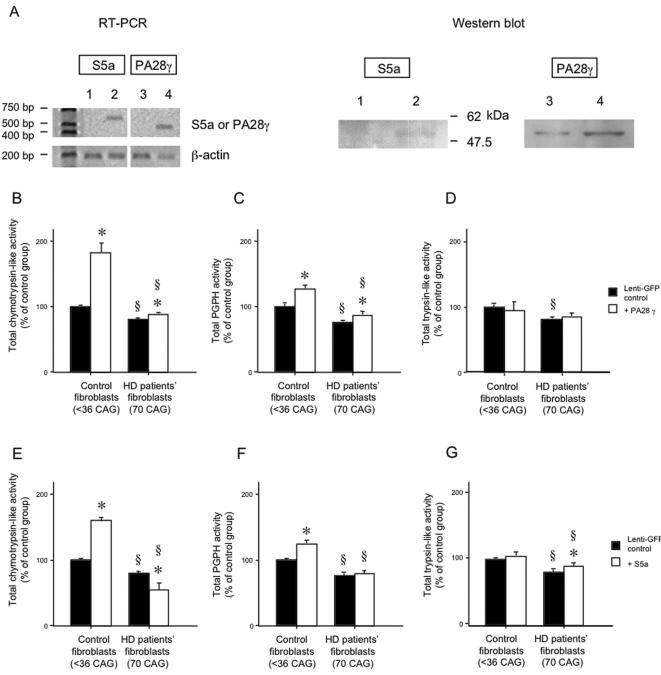
Proteasome activities following lentiviral gene transfer of PA28γ and S5a, in control and HD fibroblasts. (A) Expression levels of gene and protein of S5a and PA28γ were determined using RT-PCR and Western blot after viral gene transfer to HD fibroblasts (lane 1 and 3; lenti-GFP transduced cells, lane 2; S5a transduced cells, and lane 4; PA28γ transduced cells). (B–G) Proteasome activities were increased by lentiviral gene transduction of PA28γ. Chymotrypsin-like (B, E), PGPH (C, F) and trypsin-like (D, G) activities were detected in normal control (<36 CAG) and HD patients' skin fibroblasts, which overexpress PA28γ (B–D) or S5a (E–G). The overexpression of PA28γ increased chymotrypsin and PGPH-like, but not trypsin proteasome activities in both normal control and HD fibroblasts compared to lenti-GFP transduction. Chymotrypsin activities and PGPH activities were increased in control fibroblasts by overexpression of S5a. However, in HD patients' fibroblasts, S5a did not increase PGPH activities and slightly decreased chymotrypsin-like activities (§, p<0.05 between control and HD fibroblasts. *, p<0.05 between the gene transferred groups of control protein GFP and PA28γ or S5a). The experiments were repeated three times in triplicate.

Since HD patients predominantly show selective cell death for medium-sized spiny neurons in caudate-putamen (striatum) in late disease stages, but not in other tissues such as skin fibroblasts, it is interesting to compare the effects of proteasome activators in HD fibroblasts with HD model striatal neurons. For the HD in vitro model systems, we chose HD patients' skin fibroblasts [9], which show UPS dysfunction but are not vulnerable to the disease process. For modeling the most vulnerable cell type in HD, we used rat embryo derived striatal neurons with inducible mutant huntingtin expression [16]. These huntingtin-inducible striatal neurons expressed mutant huntingtin based on the Tet-ON-system [16] representing an early stage of targeted vulnerable cells in HD. By comparing wild type with mutant huntingtin overexpressing striatal neurons, we addressed the question of the consequences of altered proteasome function. Finally, we overexpressed proteasome activators in the HD model cells using lentiviral gene transfer (Figure 1A), and determined UPS function and cell viabilities after exposure to HD relevant toxins.

### Overexpression of PA28γ but not S5a can up-regulate proteasome activities in normal control and HD patient skin fibroblasts

In a previous study, the overexpression of PA28α and β increased chymotrypsin-like and PGPH activities in control, but not in HD patient fibroblasts [9]. In contrast, overexpression of another subunit, PA28γ, was able to significantly increase chymotrypsin-like and PGPH activities in both normal human control and HD patients' fibroblasts (Figure 1A and 1C). Nonetheless, the increase of proteasome activities in HD patients' fibroblasts was significantly smaller than those seen in normal control fibroblasts after PA28γ gene-transfer (Figure 1B–1D). We next investigated the overexpression of the S5a subunit of PA700, which is important for the recruitment of ubiquitinated abnormal proteins into the UPS [17], [18] (see introduction). While lentiviral overexpression of S5a increased chymotrypsin-like and PGPH activities in control human skin fibroblasts, this did not occur in HD patient fibroblasts (Figure 1E–1F). In fact, S5a expression increased trypsin-like activity and marginally but significantly (p<0.05) reduced chymotrypsin-like activity in HD fibroblasts. Notably, the effects of the overexpression of PA28γ and S5a had a larger impact on chymotrypsin and PGPH-like activities than on trypsin activity in both normal control and HD patients' fibroblast cells (Figure 1B–1G). In parallel experiments, gene-transfer of p58 (another subunit for PA700) did not significantly alter proteasome activity in either normal (109±18% of control, p = 0.29) or HD patient fibroblasts (112±16% of control, p = 0.23).

### Mutant huntingtin expressing striatal neurons show reduced proteasome activities

To study UPS modification in the more vulnerable cells, such as striatal neurons, we used inducible cell lines expressing the wild type (26CAG, CTRL, *control striatal neuron*) or mutant N-terminal 548 amino acid fragment of huntingtin (105CAG, Htt, *HD model striatal neuron*) [16]. As we showed in HD patients' fibroblasts, we also found that chymotrypsin-like, PGPH, and trypsin-like proteasome activities were relatively decreasded in HD model striatal neurons, compared to the control striatal neurons, at both pre-differentiation (Seo and Isacson, unpublished data) and the neuronal post-differentiation stage. Notably, control and HD model striatal neurons showed significantly increased proteasome activities [in *control striatal neurons*; chymotrypsin: 161±6% (p<0.05), PGPH-like: 143±7% (p<0.05), trypsin: 134±9% (p<0.05); in *HD striatal neurons*; chymotrypsin: 129±8% (p<0.05), PGPH-like: 123±7% (p<0.05), trypsin: 111±10% (p = 0.12)] compared to parental striatal cells (ST14A; without exogenous huntingtin transfection). These results demonstrate that overexpression of mutant huntingtin with expanded CAG repeats produces a major UPS dysfunction in HD model striatal neurons compared to control striatal neurons.

### Overexpression of PA28γ but not S5a increases proteasome activities in wild type and mutant huntingtin overexpressing striatal neurons

**Figure 2:**
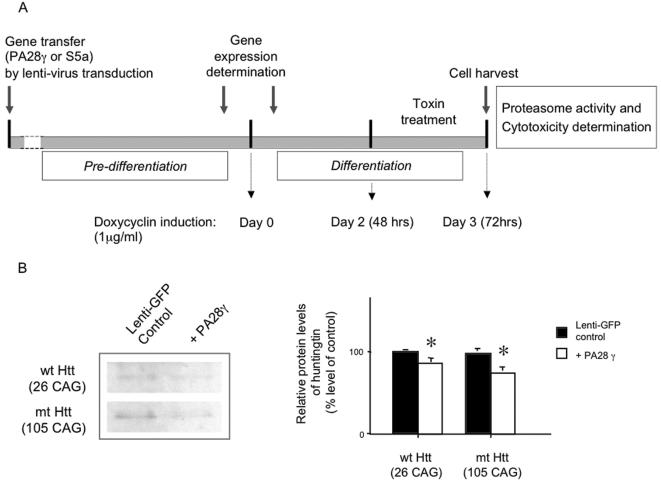
Lentiviral gene transfer of PA28γ and S5a, in control and HD model striatal neurons. (A) Schematic experimental outline of gene transfer and differentiation of striatal neurons followed by exposure to HD model experimental toxins. HD model striatal cells were gene engineered with PA28γ, S5a. After verification of expression for the transferred genes, cells were grown in medium containing 1μg/ml of doxycyclin for 48h before toxin treatment. After 24 h incubation in the toxic environment, medium was collected for the MTS assay and cells were harvested for proteasome activity determination. (B) Semiquantitative Western blot of the huntingtin showing a slight decrease of protein levels by lenti-viral transduction of PA28γ gene into HD model striatal cells. Results are shown as percentage of levels of lenti-GFP control group (* p<0.05).

To provide functional and potentially therapeutic cellular models, we next performed gene transfer of several proteasome activator subunits including the PA28α, β and γ subunits, for the 20S proteasome, and the S5a and p58 subunits of PA700 for the 26S proteasome into the HD model striatal neurons. After neuronal differentiation, we determined proteasome activities and also administered toxins that have been shown to elicit HD-like pathology in control and HD model striatal neurons (Figure 2).

**Figure 3:**
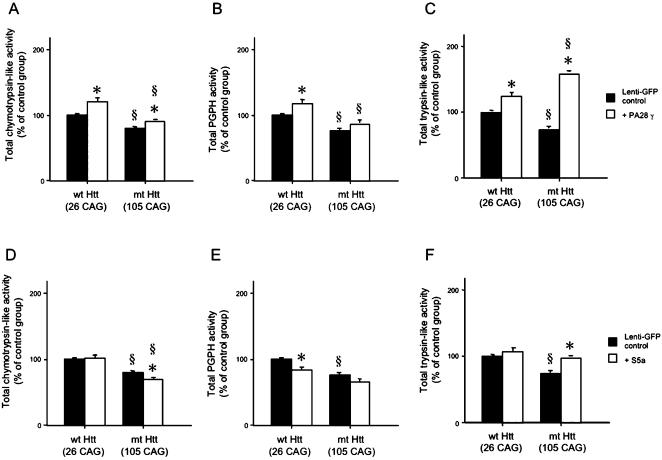
Proteasome activities following lentiviral gene transfer of PA28γ and S5a, in control and HD model striatal neurons. (A–F) Chymotrypsin-like (A, D), PGPH (B, E) and trypsin-like (C, F) activities were detected. Both wild type (CTRL, 26 CAG, control striatal neurons) and mutant huntingtin overexpressing HD model striatal neurons (Htt, 105 CAG, HD model striatal neurons) [16] were transduced with PA28γ or S5a. Basal proteasome activities were decreased in HD model striatal neurons compared to control cells with normal range of CAG repeats. However, the overexpression of PA28γ increased proteasome activities in both control and HD model striatal neurons. The overexpression of S5a decreased chymotrypsin-like activities in HD model striatal neurons and decreased PGPH activities in control striatal neurons. In contrast, total trypsin-like activities were slightly increased in both control and HD model striatal neurons after the gene transfer of S5a (§, p<0.05 between control and HD striatal neurons. *, p<0.05 between the gene transferred groups of control protein GFP and PA28γ or S5a). The experiments were repeated three times in triplicate.

Similar to the previous results in a systematic analysis in human fibroblasts [9], the viral gene transduction of PA28α and β subunits did not significantly alter the proteasome activities HD model striatal neurons in this study (for example, chymotrypsin activity, PA28α: 105±12%, p = 0.11, PA28β: 115±15%, p = 0.21), compared to lenti-GFP transduction. The gene transduction of PA p58 subunit also did not significantly alter the proteasome activities in either control (112±10% of control, p = 0.18) or HD model striatal neurons (109±12% of control, p = 0.15) compared to lenti-GFP transduction. In contrast, PA28γ subunit overexpression produced a marked effect on proteasome activities including trypsin-like, chymotrypsin-like and PGPH activities in HD model striatal neurons (Figure 3A–3C). In addition, PA28γ overexpression slightly decreased huntingtin protein levels in HD model striatal neurons after administration of doxycycline (Figure 2B). In both control and HD model striatal neurons, S5a overexpression caused only minor changes in the proteasome activities tested, (Figure 3D–3F). In fact, S5a expression slightly decreased chymotrypsin-like activity in HD model striatal neurons, and PGPH activity in control striatal neurons. Nonetheless, S5a slightly increased the trypsin-like activity in HD model striatal neurons.

### PA28γ but not S5a improves cell survival of mutant huntingtin expressing striatal neurons, which showed increased vulnerability to neuropathological toxins

**Figure 4:**
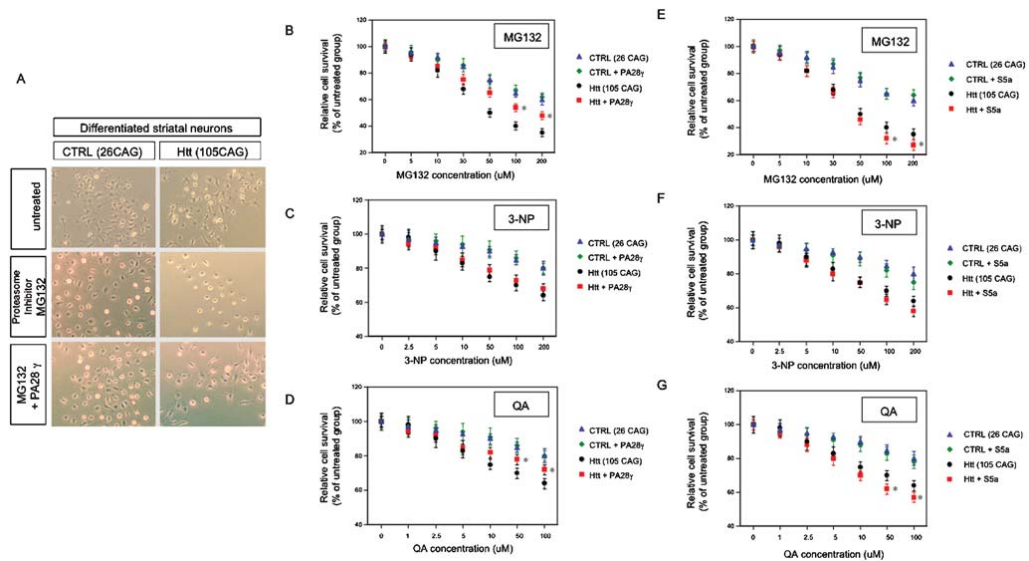
Experimental exposure of PA28γ (A–D) or S5a (E–G) overexpressing control and HD model striatal neurons to toxin modeling pathophysiological processes observed in HD. (A) MG132 treated control (CTRL, 26 CAG) and HD model striatal neurons (Htt, 105 CAG) transduced with PA28γ. Shown are cell viabilities after 24 hours of exposure to MG132. (B–G) The reversible proteasome inhibitor, MG132 (B, E); the mitochondrial inhibitor, 3-NP (C, F); and the excitotoxin, QA (D, G) were used at various concentrations to treat control and HD model striatal neurons. HD model striatal neurons showed significantly decreased the resistance to those neuropathological toxins compared to control striatal neurons. PA28γ significantly improved cell survival, and S5a significantly decreased cell survival after exposure to MG132 and QA, but not 3-NP, respectively (§, p<0.05 between wild-type and mutant huntingtin overexpressing striatal neurons. *, p<0.05 between the gene transferred groups of control protein GFP and PA28γ or S5a). The experiments were repeated three times in triplicate.

We next evaluated if overexpression of PA28γ and S5a lead to improved cell function and increased cell survival in HD model striatal neurons. We used three different HD relevant toxic stimuli: MG132 (a reversible proteasome inhibitor), 3-NP (a mitochondrial toxin) or QA (quinolinic acid, an excitotoxin). Cell viability of differentiated control or HD model striatal neurons was examined using the MTS assay, which reflects the number of viable cells [19]. There was a dose-dependent toxicity for each toxin in control and HD model striatal neurons. HD model striatal neurons (Htt, 105CAG) showed less cell viability after toxic exposure to MG132 (Figure 4A), 3-NP (data not shown) and QA (data not shown) than control striatal neurons (CTRL, 26 CAG) in the same conditions. Notably, PA28γ expression significantly improved cell survival of HD model striatal neurons (Htt, 105 CAG) compared to lenti-GFP transduction after MG132 proteasome inhibitor treatment and QA excitotoxicity (Figure 4A, 4B and 4D). However, PA28γ expression did not improve cell survival after mitochondrial toxin exposure (3-NP) (Figure 4C). Overexpression of the S5a subunit did not enhance neuronal health in either control or HD model striatal neurons (Figure 4E–4G). S5a exacerbated MG132 and QA toxin effects, but did not alter the effects of 3-NP treatment (Figure 4F). These data demonstrate that overexpression of the proteasome activator subunit PA28γ (but not S5a) can reduce HD model neuronal cell damage (and death) associated with proteasome dysfunction and excitotoxicity, but did not protect the cellular dysfunction produced by direct mitochondrial complex II toxicity.

## Discussion

In this study, we performed viral gene transduction of proteasome activator (PA) subunits (α, β, γ for PA28 and S5a and p58 for PA700) to HD patients' and control fibroblasts and to control and HD model striatal neurons, to determine the functionality of the UPS and potential therapeutics for this disease. HD model striatal neurons with mutant huntingtin expression (105 CAG) showed reduced proteasome activity and increased vulnerability to proteasome inhibition, mitochondrial complex II inhibition, and QA excitotoxicity compared to control striatal neurons expressing wild type huntingtin (26 CAG). Specifically, PA28γ enhanced proteasome activities and improved cell survival after proteasome inhibition (by MG132) and QA excitotoxicity, but not after mitochondrial inhibition (by 3-NP).

In a related study, using a conditional transgenic mouse model of HD, neuronal inclusions and HD-like symptoms were reversed when mutant huntingtin protein expression was blocked [20]. These data indicate that continuous expression of mutant huntingtin is required for the expression of HD pathology [20]. Such clearance of mutant huntingtin in neuronal cultures in HD mice depended on proteasome function given that the irreversible proteasome inhibitor lactacystin prevented the resolution of the protein aggregate [21]. Previous studies have also demonstrated that UPS inhibition decreases cell viability [22], increase polyglutamine aggregation in Spinocerebellar ataxia type 3/Machado-Joseph disease (SCA3/MJD) in vitro, and alter cell morphology [23]. Mutant huntingtin overexpressing neuroblastoma cells with ubiquitin cotransfection showed increased protein aggregates and apoptic cell death, suggesting ubiquitin involvement in HD pathological cell death [6]. Overexpression of ubiquitin E3-ligase (Siah1) increased the nuclear translocation of mutant huntingtin and cytotoxicity, indicating that ubiquitin ligation is also critical in HD pathology [24]. A shorter half-life of huntingtin delayed aggregate formation, but increased cellular toxicity [25]. Therefore it appears that cell toxicity in part is caused by unprocessed mutant huntingtin protein with the extended CAG repeats. These data imply that aggregate formation itself may not be a direct cause for the cell death, but a cellular defense mechanism. In our study, the mutant huntingtin overexpressing HD model striatal neurons did not show protein aggregates even though they demonstrated UPS dysfunction, which is similar to HD patients in early disease stages (grade 0–1)[9]. These results indicate that the mutant huntingtin itself (in the pre-aggregation stage) can produce significant proteasome dysfunction in HD. Generally, cytosolic mutant proteins may be toxic[26] to cells, and so they need to be processed by the UPS or other pathways such as lysosomes and autophagy [27]. However, when the absolute amount of cytosolic mutant protein reaches a certain limit, protein aggregations occur and consequently form nuclear inclusion bodies [5], [28]. In this scenario, aggregates are not harmful per se but just by-products in the cell. Consequently, simple aggregation inhibition may not be a proper solution for abnormal protein handling in neurodegenerative diseases [9], [29], [30].

Previously, we found significant proteasome dysfunction in the several brain regions and skin fibroblasts from HD patients [9]. However, there are likely multiple interferences by mutant huntingtin that can precipitate HD pathology in vulnerable brain regions [9]. Impaired metabolic mitochondrial complex II–III (MC II–III) energy production is also involved in the vulnerability of the neurons in caudate-putamen area of HD patients [9], [31]. Ruan et al. also found that striatal cells from mutant huntingtin knock-in mice are selectively vulnerable to MC II–III inhibition [32]. BDNF levels are down regulated in several brain regions of HD patients [9]. Finally, by excitotoxicity, the N-methyl-D-aspartate (NMDA) receptor agonist quinolinic acid (QA) destroy medium sized spiny neuron in very similar pattern to that seen in human HD pathology [33]–[35].

PA28γ overexpression in this study caused improved UPS function as assessed by known proteolytic activities of the proteasome in both HD fibroblasts and HD model striatal neurons. Critically, such PA28γ gene transduction reduced the HD relevant toxicity produced by proteasome inhibitor (MG132), and/or excessive glutamate receptor (QA) mediated activity. However, no neuroprotection was observed after mitochondrial complex inhibition (3-NP). One potential explanation for this result is that PA28γ is a non-ATP dependent regulator of the UPS [36], and therefore less connected to alterations of mitochondrial metabolism. Given that QA and NMDA induced cell death is preceeded by large increases in misfolded and ubiquitinated proteins [37], [38], the observed neuroprotection against both MG132 and QA is understandable. Whereas mitocondrial toxicity decreases the threshold for NMDA mediated toxicity to increase calcium influx to the cells [39]–[41], there is no evidence that NMDA receptor impairs mitochondrial function. The consequence of such analysis is that PA28γ could contribute to reductions in both excitotoxicity and protein toxicity whereas protection against the mitochondrial dysfunction would require an additional type of intervention. Proteasome activators of the UPS modulate different proteolytic enzyme activities by several mechanisms. This is due to the different alterations of protein structure by which the PA28 and PA700 regulators bind to the 20S proteasome core to help peptide substrates reach the catalytic core beta subunits. For example, PA28g and S5a are participating in two different major protein degradation pathways in the UPS: PA28g is involved in non-ubiquitinated protein degradation, while S5a is associated with ubiquitinated protein degradation [42]. PA28g is predominantly located in the nucleus, while S5a exists in the cytosol [42], [43]. Curiously, in our experiment, S5a overexpression decreased cell survival in the response to MG132 and QA in HD model striatal neurons. Perhaps the overexpression of S5a (one out of nine subunits complex of PA700) is not sufficient to accomplish functional effects. Alternatively, it could be a stoichiometric problem, in which there is unbalanced competition to form the 26S proteasome complex [7]. Yet another problem with excess amounts of free S5a may be the inhibition of proteasome degradation by sequestering polyubiqutinated substrates from reaching the proteasome [17], [44].

It was recently reported that genetic reduction of PA28γ (also denoted REGγ) did not alter the pathological phenotype or inclusion body formation in the striatum of R6/2 HD mice [45]. We think this absence of neuroprotection by PA28γ compared to our positive findings can be explained by differences of experimental approaches and HD models used. For example, R6/2 mice always show *increased* proteasome activities in the striatum [46], which therefore does not model the UPS dysfunction seen in HD patients' brains and HD fibroblasts [9]. However, HD transgenic mice with full-size mutant huntingtin expression (YAC72 transgenic mice) do show UPS inhibition resembling that seen in HD patients [46]. This at least indicates that R6/2 transgenic mice do not model mature HD pathology due to abnormal protein processing in the UPS. Moreover, the HD R6/2 transgenic mice also completely lack QA-induced striatal excitotoxicity in modeling HD pathology [47]. Consequently, the lack of additional HD pathology by knock out of PA28γ function during development of R6/2 mice [45] may not represent a challenge to our findings of neuroprotective effects by PΑ28γ enhanced UPS function.

In summary, these results demonstrate a role of mutant huntingtin in protein toxicity and specifically provide therapeutic targets and candidates for gene transfer to enhance proteasome function. Although proteasome dysfunction is probably only one of multiple factors involved in the dynamic and progressive disease process of HD [9], our data at least demonstrate that proteasome activators are relevant candidates for future comprehensive and effective treatment approaches to HD.

## Materials and Methods

### Experimental Design

In the experiments, we performed gene transfer of several proteasome activator subunits including PA28α, β and γ, for the 20S proteasome, and S5a and p58 subunits of the PA700 for the 26S proteasome into normal and HD patient's skin fibroblasts, and control and HD model striatal cells. After determination of the expression of the transduced genes using RT-PCR and Western blot (Figure 1A), cells were differentiated as previously described [16] (Figure 2). Cell cultured medium was collected for MTS cytotoxicity test and cells were harvested for proteasome activity determination. In addition, we administered HD relevant toxins at various concentrations for 24 hours for cytotoxicity study.

### Construction of Lentiviral Vectors and Lentiviral Transduction of proteasome activators, PA28γ and S5a

The lentivirus vector system used in our studies was kindly provided by Drs R. Zufferey and D. Trono, University of Geneva, Switzerland [48]. The PA28α, β, γ p58 and S5a cDNAs were kindly provided by Dr. Rechsteiner (University of Utah [14], [43]) and Dr. Tanaka [42], and cloned into pRRL.cPPT.PGK.GFP.W.Sin-18 (Lenti-eGFP) replacing the eGFP with the PA28γ or S5a genes. Fibroblasts and striatal cells were transduced with Lenti-eGFP as a transduction control, PA28γ or S5a at a multiplicity of infection (MOI) of 5 to 20 as previously described [9].

### Cell Culture

Normal and HD human fibroblasts used in this study were obtained from the Coriell Cell Repository ([9] GM08399, GM04689, GM04732). Fibroblasts were cultured in minimum essential medium (MEM; Gibco, Carlsbad, CA) supplemented with 15% fetal bovine serum (Hyclone, Logan, UT), 2 mM L-glutamine (Gibco), 0.1 mM nonessential amino acids (Gibco), penicillin and streptomycin. Huntingtin inducible striatal cells were cultured at 37°C and 33°C in DMEM, supplemented as described previously [16], [49]. The expression levels of huntingtin protein were determined after administration of doxycycline (1 µg/ml) for 24 hours using Western blot analysis (Figure 2B).

MG132 (Calbiochem), 3-NP (Sigma), QA (Sigma) were used at the indicated concentrations in the cell cultures. After 24 hours of treatment, medium was collected to determine cell viability by a quantitative colorimetric assay, the modified MTS assay (Promega). Harvested cells were lysed in 50 mM Tris pH8.0, 150 mM NaCl, 5 mM EDTA, 1% Triton X-100, 10 µg/mL aprotinin, 25 µg/mL Leupeptin, 10 µg/mL Pepstatin, 1 mM PMSF; all protease inhibitors purchased from Sigma). Homogenates were centrifuged at 14,000× g for 30 min at 4°C. The protein levels were determined using the Bio-Rad Protein Assay (BIO-RAD, Hercules, CA). Samples containing equal amounts of total protein were used for the determination of proteasome activities and Western blots.

### Reverse-transcriptase (RT) PCR for transfered gene validation

RNA samples were extracted from harvested cells using Tri Reagent (Sigma, St. Louis) and were reverse transcribed into cDNA using the Superscript™ reverse transcriptase kit (Invitrogen, Carlsbad, CA) and oligo(dT)_20_ as primers. Reverse transcriptase reaction were carried out at 50°C, for 50 min. Synthesized cDNA template were amplified with forward primers for S5a (5′-ATCTATGGAAGAGCAGCGG-3′) and PA28 γ (5′-GGGTACAGCTCCTGATTCC-3′) and the virus backbone reverse primer, WPRE (5′-AGCAGCGTATCCACATAGC-3′). Human β-actin primers (forward: 5′-GGCGGCAACACCATGTACCCT-3′; reverse: 5′-AGGGGCCGGACTCGTCATACT-3′) were used to determine cellular RNA expression level. PCR was performed with G-*Taq* DNA polymerase (Labopass, Seoul, Korea) with a denaturing step for 2 min at 94°C, followed by 40 cycles of 1min at 94°C, 1 min at 58°C, and 2 min at 72°C, and terminated by an elongation step at 72°C for 7 min. PCR products were visualized in 1% agarose gel electrophoresis.

### Determination of proteasomal function

Using synthetic peptides, three different proteolytic activities were measured to detect proteasome activities: (1) chymotrypsin-like (after hydrophobic residue), (2) trypsin-like (after basic residues), or (3) PGPH (after basic residues) activities [13]. These different catalytic activities are due to the function of different subunits of the catalytic core. In this study, proteasome function was determined by continuously measuring the fluorescence of 7-amido-4-methylcoumarin (AMC) (excitation 380 nm, emission 460 nm) generated from peptide-AMC linked substrates [50]. Reactions were conducted in a final volume of 200 µl containing 50 mM Tris-HCl buffer (pH 7.5) and 1 mM EDTA. After adding samples to the reaction mixtures, reactions were initiated by the following substrates: Suc-Leu-Leu-Val-Try-AMC (65 µM) for chymotrypsin activity, Z-Leu-Leu-Glu-AMC (75 µM) for PGPH activity and Boc-Leu-Arg-Arg-AMC (71 µM) for trypsin-like activity. Reactions were followed for 240 min at 25°C and enzymatic activities were expressed as fluorescence units (FU)/min/mg protein.

### Western blot

The protein expression levels were determined from the cell extract using specific antibodies as previously described [9]: PA28γ (Calbiochem 1∶2,500), S5a (Calbiochem, 1∶1,000), huntingtin (Chemicon, 1∶5,000). Quantification of immunoreactive bands was performed using densitometry. The results were confirmed by duplicate measurements of the same samples.

### Statistical analysis

All statistical analyses were carried out using JMP (version 3.1.6, SAS Institute). Data were objectively compared between different groups at different stages of disease using unpaired Student's t test and 2-way ANOVA followed by Turkey-Kramer posthoc analysis.
